# Alkaline pH Is a Signal for Optimal Production and Secretion of the Heat Labile Toxin, LT in Enterotoxigenic *Escherichia Coli* (ETEC)

**DOI:** 10.1371/journal.pone.0074069

**Published:** 2013-09-18

**Authors:** Lucia Gonzales, Zahra Bagher Ali, Erik Nygren, Zhiyun Wang, Stefan Karlsson, Baoli Zhu, Marianne Quiding-Järbrink, Åsa Sjöling

**Affiliations:** 1 Department of Microbiology and Immunology, Institute of Biomedicine, University of Gothenburg, Gothenburg, Sweden; 2 Instituto de Biología Molecular y Biotecnología, Universidad Mayor de San Andrés, La Paz, Bolivia; 3 CAS Key Laboratory of Pathogenic Microbiology and Immunology, Institute of Microbiology, Chinese Academy of Sciences, Beijing, PR China; Charité-University Medicine Berlin, Germany

## Abstract

Enterotoxigenic *Escherichia coli* (ETEC) cause secretory diarrhea in children and travelers to endemic areas. ETEC spreads through the fecal-oral route. After ingestion, ETEC passes through the stomach and duodenum before it colonizes the lower part of the small intestine, exposing bacteria to a wide range of pH and environmental conditions. This study aimed to determine the impact of external pH and activity of the Cyclic AMP receptor protein (CRP) on the regulation of production and secretion of heat labile (LT) enterotoxin. ETEC strain E2863wt and its isogenic mutant E2863ΔCRP were grown in LBK media buffered to pH 5, 7 and 9. GM1 ELISA, cDNA and cAMP analyses were carried out on bacterial pellet and supernatant samples derived from 3 and 5 hours growth and from overnight cultures. We confirm that CRP is a repressor of LT transcription and production as has been shown before but we show for the first time that CRP is a positive regulator of LT secretion both *in vitro* and *in vivo*. LT secretion increased at neutral to alkaline pH compared to acidic pH 5 where secretion was completely inhibited. At pH 9 secretion of LT was optimal resulting in 600 percent increase of secreted LT compared to unbuffered LBK media. This effect was not due to membrane leakage since the bacteria were viable at pH 9. The results indicate that the transition to the alkaline duodenum and/or exposure to high pH close to the epithelium as well as activation of the global transcription factor CRP are signals that induce secretion of the LT toxin in ETEC.

## Introduction

Diarrhea caused by Enterotoxigenic *Escherichia coli* (ETEC) remains a major cause of mortality and morbidity worldwide. ETEC is a leading cause of infectious diarrhea in young children in developing countries and represent the principal etiological agent of traveleŕs diarrhea [1], [2], [3]. Many strains of ETEC express a virulence factor called heat-labile enterotoxin or LT [2]. LT is a multimeric A-B5-type toxin, consisting of a single A subunit, LTA, and a ring of five B subunits, LTB. LTB mediates the toxińs binding properties and LTA ADP ribosylates host G proteins [4], [5]. LTA acts on Gsα, a protein that governs the activity of adenylate cyclase in eukaryotic cells. The ADP ribosylation of Gsα results in the overproduction of cyclic AMP (cAMP), which causes increased chloride secretion and disrupts sodium absorption [4]. Water is lost to the intestinal lumen as a consequence of these alterations of ion transport [6]. Each subunit of LT is translated separately from a bicistronic message and then transported to the periplasm, where holotoxin assembly either might occur spontaneously [7], [8] or with the aid of the disulfide bridge forming protein DsbA [9], [10], [11].

ETEC strains differ in their ability to produce and secrete the LT toxin and typically retain most of its produced LT toxin in the periplasm or associated to the LPS structures on the outer membrane [12], [13], [14], [15]. The expression of the heat labile toxin LT is known to be repressed at the level of transcription by cyclic AMP repressor protein CRP [16]. In absence of glucose in the culture media, the levels of cytoplasmic cAMP rise and activate CRP binding. Thus, LT gene expression is turned on in the presence of glucose when cAMP levels decrease, although different studies have shown that the secretion of LT toxin is either positively or negatively regulated by the addition of glucose to the medium [16], [17], [18]. LT has been reported to be secreted either as a free toxin [14] or associated with outer membrane vesicles [12], [13], [15].

In order to colonize the human gastrointestinal tract, *E. coli* must be able to grow between pH 4.5 and pH 9 [19]. Over this wide pH range, *E. coli* preserves enzyme activity, as well as protein and nucleic acid stability, by maintaining the cytoplasmic pH in the range of 7.2 to 7.8 [20], [21]. Although the cytoplasm is maintained at near neutral pH, the periplasm of gram-negative bacteria is exposed to the extracellular environment and essentially maintain the same proton concentration as the surrounding environment [22]. The LT holotoxin is assembled in the periplasm and hence exposed to external pH which may thus implicating that pH might affect LT assembly and secretion. In addition, the role of pH on the transcriptional regulation of *eltAB,* which encodes the LT toxin, is not known. In this study, we assessed the production and secretion of LT toxin under different pH conditions and how CRP influence net LT output.

## Materials and Methods

### Ethical Statement

All animals in the study were treated and housed under specific-pathogen-free conditions in accordance to the Swedish Animal Welfare Act (1988∶534) and the Animal Welfare Ordinance (1988∶539). Approval for the study was given by the Ethical Committee for Laboratory Animals in Gothenburg, Sweden (ref no. 227–2012).

### Plasmids and Strains

Mutagenesis of the *crp* gene was performed in two strains; E2963 (LT CS6) and H10407 (LT STh STp, CFA/I). Mutagenesis of the ETEC strain E2863 was performed by making an in-frame deletion that removed most of the codons, using a procedure described previously [23], [24], [25]. Briefly, a flanking sequence of the 5′region of *crp*, including several nucleotides of the coding region and the *OsmC* gene upstream of *crp* was PCR amplified with the primers cpr1F (*OsmC* gene upstream of *crp*) and crp2R (beginning of *crp*). The primers crp3F (end of *crp*) and crp4R (beginning of the integral membrane protein, YccS/YhfK family) were used to amplify several nucleotides of the 3′region of the gene plus part of the downstream flanking sequence. The two PCR products were annealed by an overlapping region of primer crp2R and crp3F and amplified by PCR to produce a single DNA fragment, using the outer primers (cpr1F and crp4R) (Table S2). Amplified bands were cut out from the gel and then purified using PCR Purification Kit (Qiagen, Hilden, Germany). The resulting PCR product, lacking most of the coding sequence of the *crp* gene, was digested with Bcul enzyme and ligated into a similarly digested PMT-suicide1SacB which have the *sacB* gene that produce the toxic sugar levan in the presence of sucrose in the media and a chloramphenicol resistance gene. PMT-1SacB::*crp* was introduced into the auxothrophic E. coli strain S-171 by electroporation. The donor, *E. coli* S-171 containing the plasmid PMT-1SacB::*Δcrp*, was used for conjugal transfer to Enterotoxigenic E. coli strain E2863 (LT,CS6). The donor and recipient were mixed onto M9 agar plates with chloramphenicol and grown overnight. Individual colonies were picked and grown in liquid M9 medium without chloramphenicol for 6 hours. The cultures were then diluted 100-fold in the same medium and grown for 6 hours, the procedure was repeated three times. Cultures were streaked onto LB plates that contained 6% sucrose and incubated at 37°C. Several colonies were purified from the plates, tested for chloramphenicol sensitivity, and then analyzed for the deletion using colony PCR.

The crp deletion mutant of H10407 (H10407ΔCRP*)* was constructed by a modified form of *lambda red* recombination, as described previously [26]. Briefly, a 459 bp PCR product upstream (crp/up) and 323 bp PCR product downstream (crp/down) of *crp* were independently amplified with the primer pairs crpF1′/crpF2′, and crpF3′/crpF4′. The products were combined into pET22b (Novagen, Germany, Ap^r^) containing the *kan* gene. H10407 carrying pKD46 containing the *red* recombinase genes were grown at 30°C in the presence of 10 mM arabinose in order to induce the recombinase genes and then transformed with the gel-purified crp/up-Kan-crp/down PCR products amplified with crpF1′ and crpF4′ primers. Recombinants were selected from LB plates containing kanamycin. The *crp* rescued strain was constructed by transformation of the pAK-crp plasmid into the *crp* deletion strain without the *kan* gene [27].

### Growth Conditions

The wild-type and mutant strains were grown in LB agar plates supplemented with X-gal and IPTG. Wild-type colonies were blue and mutant colonies were white and smaller thereby confirming the deletion of a functional *crp*, all new experiment were checked using blue-white screening to confirm deletion of *crp*. Cultures were routinely prepared in LBK broth only (10 g Tryptone, 5 g yeast extract, 6.4 g KCl) or contained a pH-appropriate sulfonate buffer at 100 mM. Buffers used included 2-(*N*-morpholino) ethanesulfonic acid (MES), piperazine-N,N9-bis-(2-ethanesulfonic acid) (PIPES), and 3-[(1,1-dimethyl-2-hydroxyethyl)amino]-2-hydroxypropanesulfonic acid (AMPSO). Media were adjusted for pH with KOH, to avoid high concentrations of sodium ions, which inhibit growth at high pH. ETEC strains were grown to exponential growth phase for 3 hours in LBK media (180 rpm, 37°C). Bacterial culture densities at exponential phase were measured at OD_600_ and the number of bacteria per ml culture was calculated assuming that OD_600_ 0.8 equals 1×10^9^ bacteria for the wild-type and OD_600_ 0.8 ≡ 5×10^8^ bacteria for the mutant. The OD_600_ values used to determine number of bacteria per ml were initially calculated by 10-fold serial dilutions of initial cultures set to OD_600_ = 0.8_,_ followed by CFU counting. For each experiment, the same amount of starting culture (10^7^ or 10^8^ bacteria per ml medium) was transferred into a different culture media with the respective growth requirements. For the pH influence analysis, LBK was adjusted to pH 5 with MES, pH 7 with PIPES or pH 9 with AMPSO. OD_600_ was measured and the number of bacteria/ml was calculated as described above. Samples were collected for ELISA analysis and RNA extraction at 2, 3, 4 or 5 hours or in over night cultures. The protein and gene expression analyses were calculated to reveal the protein concentrations or number of transcripts present in the initial 1 ml of culture and the results were expressed as the protein concentration or number of transcripts per bacterium.

### GM1 ELISA Quantification of CTB/LTB

Determination of LT concentration in the culture was carried out in overnight cultures by GM1 ELISA quantification of CTB/LTB as previously described [28]. LT concentrations in the samples were determined using purified r-CTB. Regression analysis (R^2^>0.98) was used to generate a standard curve used for determination of LT concentrations in the tested samples. OD_600_ of each culture was registered to determine the number of bacteria per ml and 1 ml of culture samples were collected and centrifuged for 5 min. at 13.000 g. Culture supernatant was saved for determination of secreted LT. For each sample the concentrations of LT per ml pellet and supernatant samples determined by GM1-ELISA were divided by the number of bacteria per ml in the culture as determined by OD_600_ to calculate the LT concentration per 10^9^ bacteria. For total production analysis the pellet was resuspended in its respective media followed by sonic disruption to release bacteria-bound toxins. The total production was calculated by summing the LT concentrations in the pellet and supernatant, whereas the percentage of LT secretion was determined by the ratio of LT concentration in the supernatant divided by the total production of LT.

### RNA Extraction and Real Time Reverse Transcriptase PCR

RNA extraction was performed by the RNeasy Kit (Qiagen, Hilden, Germany) and a one-column DNAse protocol (Qiagen) included to remove genomic DNA. Extracted RNA was analyzed on an agarose gel to determine integrity and absence of DNA and the concentration was measured using a Nanodrop spectrophotometer (NanoDrop Technologies, Wilmington, DE, USA). cDNA was prepared from 1000 ng RNA from each sample using the QuantiTect cDNA kit (Qiagen) which includes an extra DNAse step. A negative control without reverse transcriptase (-RT) prepared in parallel with the cDNA from the same amount of RNA and subjected to the same extra DNAse step was used in the real time RT-PCR assays to determine absence of genomic DNA in the corresponding cDNA sample. The final volume of the cDNA samples and the –RT controls was 20 µl and both were stored at −20°C until analysis. Real time RT-PCR was performed using primers specific for the *eltAB* (LT) gene [29]. A standard curve used for quantification was generated by PCR amplification using the primers and the DNA of a toxin positive ETEC strain as template. The PCR product was purified by the QiaQuick PCR purification kit (Qiagen), and the concentration was determined on the Nanodrop spectrophotometer. The PCR product copy number was determined as described previously [30] and ten-fold serial dilutions from 5×10^7^ to 5 copies/µl were prepared and stored at −20°C until use. The real time RT-PCR assays were run in 20 µl reactions using standard conditions for the ABI 7500 (Applied Biosystems Foster city, CA, USA) using 20 ng cDNA or 2 µl standard curves DNA, 8 pmol of each primer and Power SYBR®Green PCR Master Mix (Applied Biosystems). The levels of transcripts were first determined as the number of transcripts per 20 ng of total RNA converted into cDNA and the absolute numbers of transcripts in the original 1 ml sample were determined by considering the RNA extraction volumes and the volume used for cDNA synthesis. The level of transcription in each sample was obtained by dividing the number of transcripts in the original sample by the number of bacteria as determined by OD_600_ and expressed as number of transcripts per bacterium.

### cAMP Analysis

cAMP levels were measured in the pellet and supernatant of bacteria grown for 3 h in LBK media only and in media adjusted to pH 5, 7 and 9, by the Direct cyclic AMP Enzyme-linked Immunosorbent Assay, ELISA (Enzo, life Sciences) according to the manufacturer instructions. The data was handled utilizing a 4 parameter logistic (4 PL) curve fitting program.

### Flow Cytometry Assessment of Bacteria Membrane Integrity

Membrane integrity of the bacteria grown at different pH conditions and at different time points was assessed using the Live-Dead BacLightTM (Molecular Probes Eugene, OR, USA) as described by the manufacturer. This bacterial viability kit consists of two nucleic acid stains. Green fluorescent SYTO 9 that is cell-permeable and can freely enter *E. coli*, either live or dead. In contrast, red fluorescent propidium iodide (PI) can only enter membrane-comprised cells. In our set-up 10 µl of the treated bacterial cell suspension was added to 997 µl of sterile saline. These samples were immediately stained with 3 µl of a mixture of SYTO 9 (5 µM final concentration) and PI (30 µM final concentration) and incubated for 15 min in the dark at room temperature. Flow cytometric measurements were performed immediately thereafter, using a BD FACSCalibur 4-color dual laser flow cytometer. All data on the percentages of live (L), compromised (C) and dead (D) bacteria, all together approaching 100% were acquired and processed using Flow cell software (Becton, Dickinson and Company, Franklin Lakes, NJ, USA).

### Infant Mice Model

Outbred Swiss CD1 mice were purchased from Charles River Laboratories (Sulzfeld, Germany) and used for breeding at 10 weeks of age. The capacity to persist in the infant small intestine, i.e. the colonization potential, of the wild-type ETEC strain (E2863) and the isogenic Δcrp-mutant were compared either in competition experiments or side by side and performed in principle as described previously [31]. Three to four day old CD1 mice (2.4–2.7 g) were removed from their mothers and kept at 26°C for approximately 6 hours prior to inoculation to allow emptying of stomach contents. Subsequently the pups were inoculated intragastrically with PBS-suspensions of the two strains using a standard smooth-tipped hypodermic mouse feeding needle. The dose (10^6^ bacteria per strain) was confirmed retrospectively by spreading the inoculum in duplicates on Xgal-IPTG plates. Infected mice were kept on sterile tissue paper in plastic containers at 26°C. After 3 and 18 hours groups of mice (8 in each group) were sacrificed and the entire small intestine excised and divided in two parts (upper and lower half). Following estimation of pH the tissue was homogenized in 2 ml of PBS. Suitable 10-fold serial dilutions of the resulting suspensions were plated onto blood plates for determination of CFUs. Suitable 10-fold serial dilutions of the resulting suspensions were again plated in duplicates on Xgal-IPTG media for determination of CFUs. In experiments aiming at determination secreted LT by GM1-ELISA the small intestinal tissue samples were instead collected directly into ice-cold PBS, cut open using surgical scissors, vortexed to release the luminal content and centrifuged at 16000 g in a table top centrifuge to remove bacteria and debris. Collected supernatants were stored at −80°C until use.

### Statistical Analyses

Statistical analyses were carried out with Students t-test and non-parametrical non-paired Mann-Whitney test for repeated measurements using the GraphPad Prism version 4.00 for Windows (GraphPad Software, San Diego CA, USA). P values <0.05 was considered statistically significant.

## Results

### CRP is a Negative Regulator of Production but a Positive Regulator of Secretion of LT Toxin

**Figure 1:**
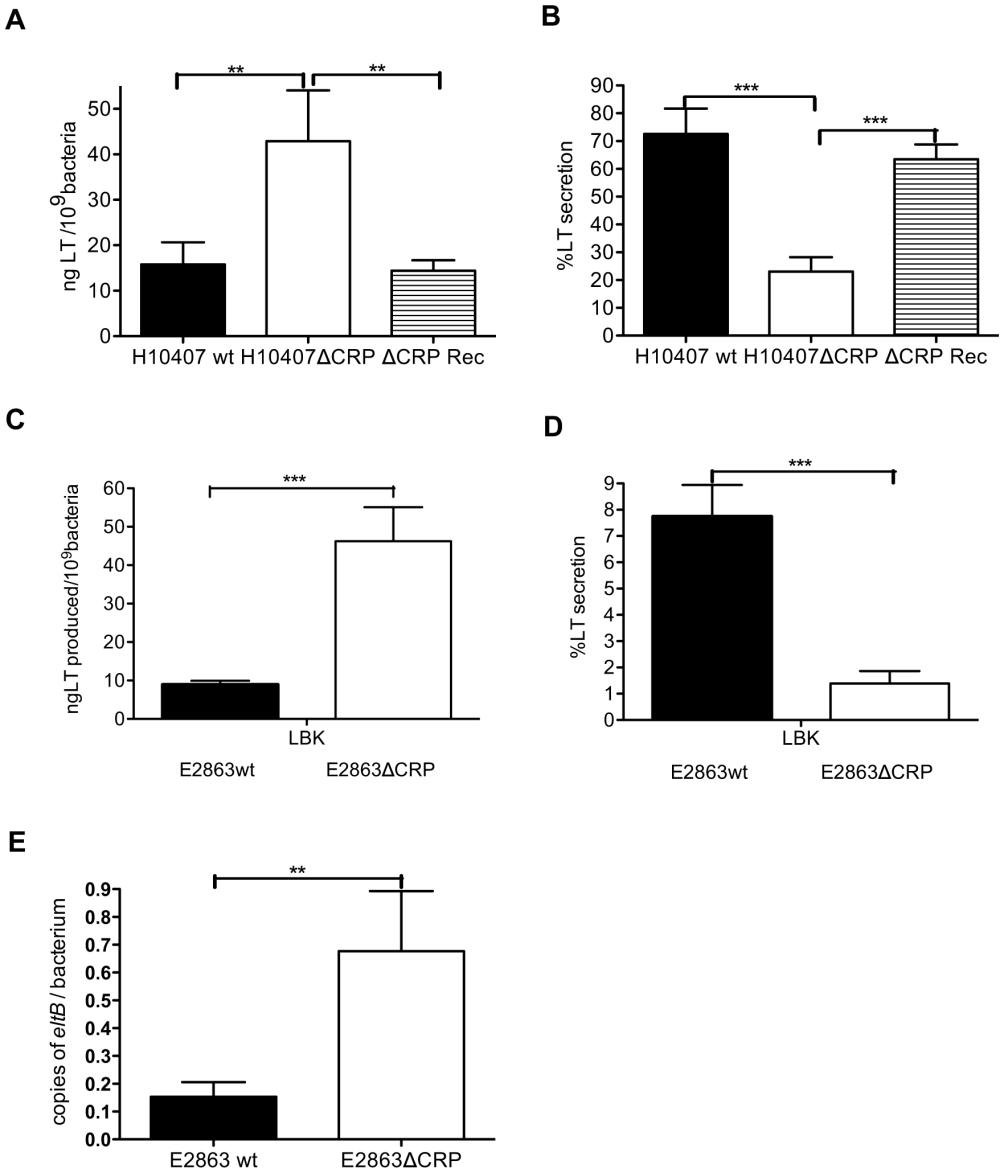
Regulation by CRP of the production and secretion of LT toxin. H10407 wild-type strain (H10407wt), cAMP receptor protein deficient strain (H10407ΔCRP), the crp complemented strain (ΔCRP-Rec), E2863 wild-type strain (E2863 wt) and cAMP receptor protein deficient strain (E2863ΔCRP) were cultured in LBK medium aerobically at 37°C. The concentrations of LT toxin were determined by GM1-ELISA in the pellet and supernatant fractions after 3 and 5 hours of growth and from overnight culture and results were pooled. Bars represent the mean and error bars represent the standard deviations of 9 independent experiment. Total production was defined as the total LT present in the pellet and supernatant and secretion as the ratio of LT supernatant to total LT. The number of transcripts of *eltAB* gene that encode LT toxin was quantified by q-PCR. A Student t test was used to calculate P values. *P<0.05, **P<0.01, ***p<0.001 A) Total production of H10407. B) Percentage of secretion of H10407. C) Total production of E2863 D) Percentage of secretion of E2863 E) Transcription of *eltAB* gene of E2863.

The expression of heat labile enterotoxin LT has convincingly been shown to be repressed at the level of transcription by the cyclic AMP receptor protein CRP in the main laboratory ETEC strain H10407 [16]. However since glucose, which leads to lower cAMP and less CRP activation, has been shown to either activate or repress LT secretion, the effect of CRP on the secretion of the toxin is not fully understood. To investigate both LT production and secretion regulated by CRP, LT toxin was measured by GM1 ELISA in the LT/STh/STp-expressing strain H10407wt, the isogenic H10407ΔCRP and the corresponding CRP complemented recombinant strain ΔCRP Rec grown in unbuffered LBK media. In order to determine whether CRP regulation was the same in different strains we also analyzed LT production and secretion in the LT expressing strain E2863wt and E2863ΔCRP grown in unbuffered LBK media. In addition, quantification of the *eltAB* mRNA that encode LT toxin was performed by q-PCR to verify that transcription of LT toxin is regulated by CRP. LT total production was higher in H10407ΔCRP than in H10407wt (p = 0.0118) and the recombinant CRP complemented strain ΔCRP Rec (p = 0.0140) (Fig. 1A). In contrast, the percentage of produced LT that was secreted were higher in H10407wt and ΔCRP Rec compared to H10407ΔCRP (p = 0.009 and 0.003, respectively) (Figure 1B). We could observe the same pattern in the E2863 strain. Production of LT was higher in E2863ΔCRP compared to E2863 wt (p<0.001) (Figure 1C) and the secretion percentage was higher in E2863 wt compared to E2863ΔCRP (p<0.001) (Figure 1D); showing that the effect of CRP on the production and secretion of LT toxin is the same in these strains. In addition, the number of LT transcripts was higher in E2863ΔCRP compared to E2863wt (p = 0.0282) (Figure 1E), confirming that CRP is a negative transcriptional regulator of the *eltAB* gene. Taken together the results confirmed that CRP is a negative regulator of LT production through transcriptional regulation of the *eltAB* gene, but showed that CRP is a positive regulator of LT secretion.

### LT Toxin Transcription, Translation and Secretion is Induced in Exponentially Growing Cells

**Figure 2:**
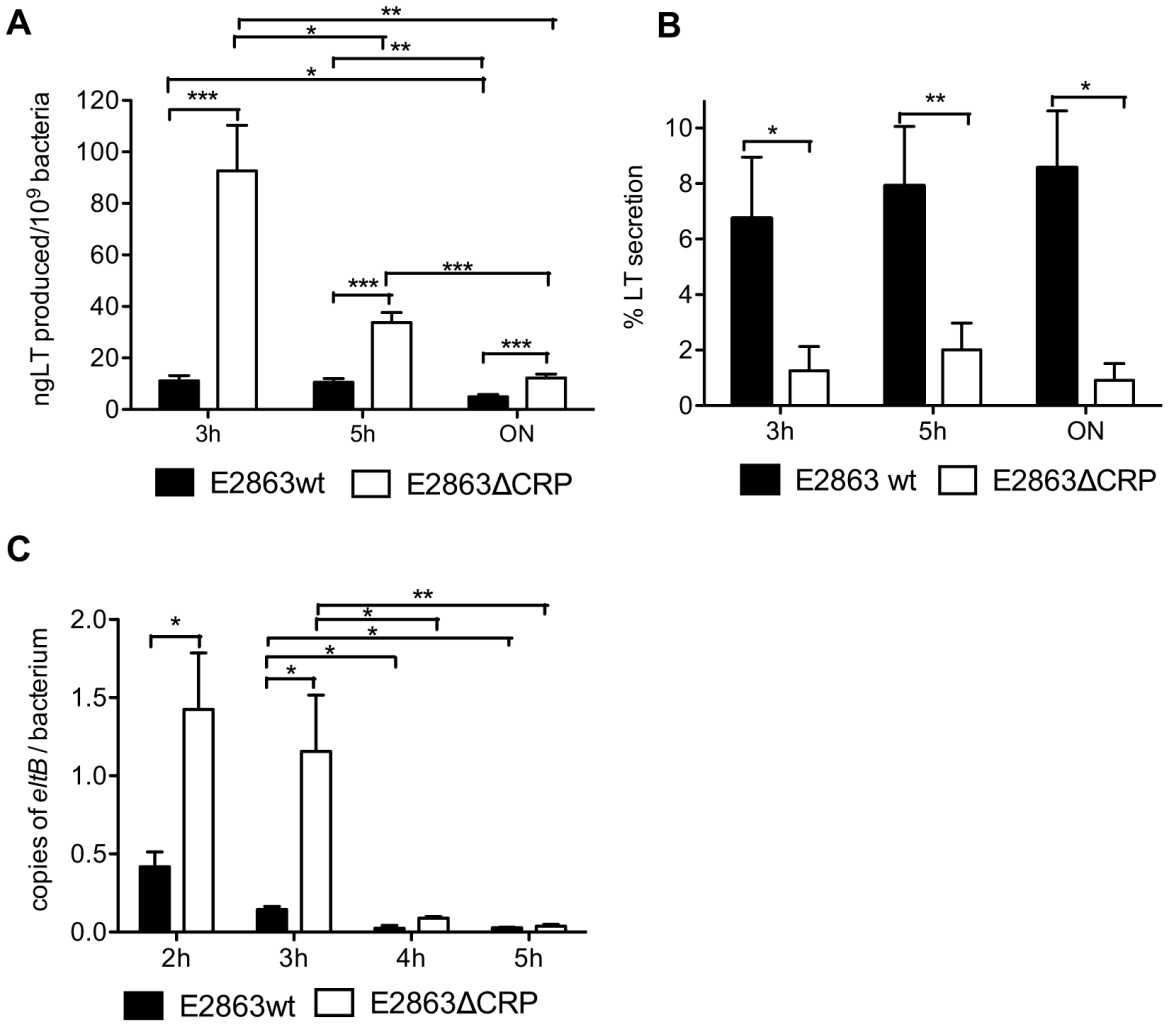
Production and transcription of the LT toxin depended on CRP is higher at early hours of the bacteria growth. E2863 wt and E2863ΔCRP were growth in LBK media and samples were taken at 3, 5 hours and from overnight cultures. GM1 ELISA was used to measure the total production and secretion of LT toxin. Expression of the toxin was quantified in *eltAB* gene for LT toxin by q-PCR. Bars represent the mean and error bars represent the standard deviations of n = 9 (ELISA) and n = 3 (q-PCR). A Student t test was used to calculate P values, *P<0.05, **P<0.01. A) Total production. B) Percentage of secretion C) Number of transcripts of *eltAB* gene.

The decay rates of various messages are affected differently by changes in the rate of cell growth [32]. Transition from exponential to stationary growth phase induces *e.g* rpoS expression and a drastic change in gene expression that can regulate virulence genes [33]. To investigate if there was an effect on the production, secretion and transcription of LT toxin at different time points in the bacteria growth curve, we analyzed LT toxin in E2863ΔCRP and E2863wt by GM1 ELISA to determine the production and secretion and by q-PCR to measure the transcription of *eltAB* gene at 3 hours for the late exponential phase, 5 hours for the initial stationary phase and from overnight culture for the stationary phase. The E2863ΔCRP strain grew considerably slower than the E2863wt so the levels of LT toxins were expressed per bacterium, based on O.D_600_. The production of LT per bacterium in E2863ΔCRP was always higher compared to E2863wt (3 h, p = 0.003; 5 h, p<0.0001 and ON, p = 0.0006), as reported above (Fig. 2A), and levels of LT production in E2863ΔCRP were especially high at 3 and 5 hours compared to overnight (p = 0.0019 and 0.0007, respectively). The E2863wt strain also had higher levels of LT production after 3 and 5 hours compared to overnight growth (p = 0.0062 and p = 0.0108, respectively) (Figure 2A). The results confirmed that LT is expressed optimally in exponential growth phase in both ΔCRP and wt strains.

In contrast to LT production, LT secretion was always higher for E2863wt than E2863ΔCRP (3 hours (p = 0.0353), 5 hours (p = 0.0025) and overnight (p = 0.0326)), confirming that CRP is a positive regulator of secretion. No difference on the percentage of secretion was found between time points in any of the strains (Figure 2B). We cannot rule out accumulation of secreted LT in the medium in the latter time points but the data indicate that secretion of LT is induced already in exponential phase. Analysis of the number of *eltAB* transcripts showed higher number of transcripts per bacterium in E2863ΔCRP than E2863wt at the initial two hours of growth (2 h, p = 0.0487 and 3 h, p = 0.0316) but not after 4 and 5 hours. Differences in the number of *eltAB* transcripts for both E2863wt and E2863ΔCRP were observed between 3 and 4 hours (p = 0.0416 and p = 0.0353, respectively) and between 3 and 5 hours (p = 0.0362 and p = 0.0021, respectively) suggesting that higher transcription levels of LT toxin occurs at early hours (Figure 2C). Taken together, the results show that LT production and secretion is induced at early hours in exponential phase in both the wild-type and the CRP mutant.

### LT Secretion is pH Dependent and Increases with Alkalinity Resulting in Maximal Production and Secretion Levels at pH 9

**Figure 3:**
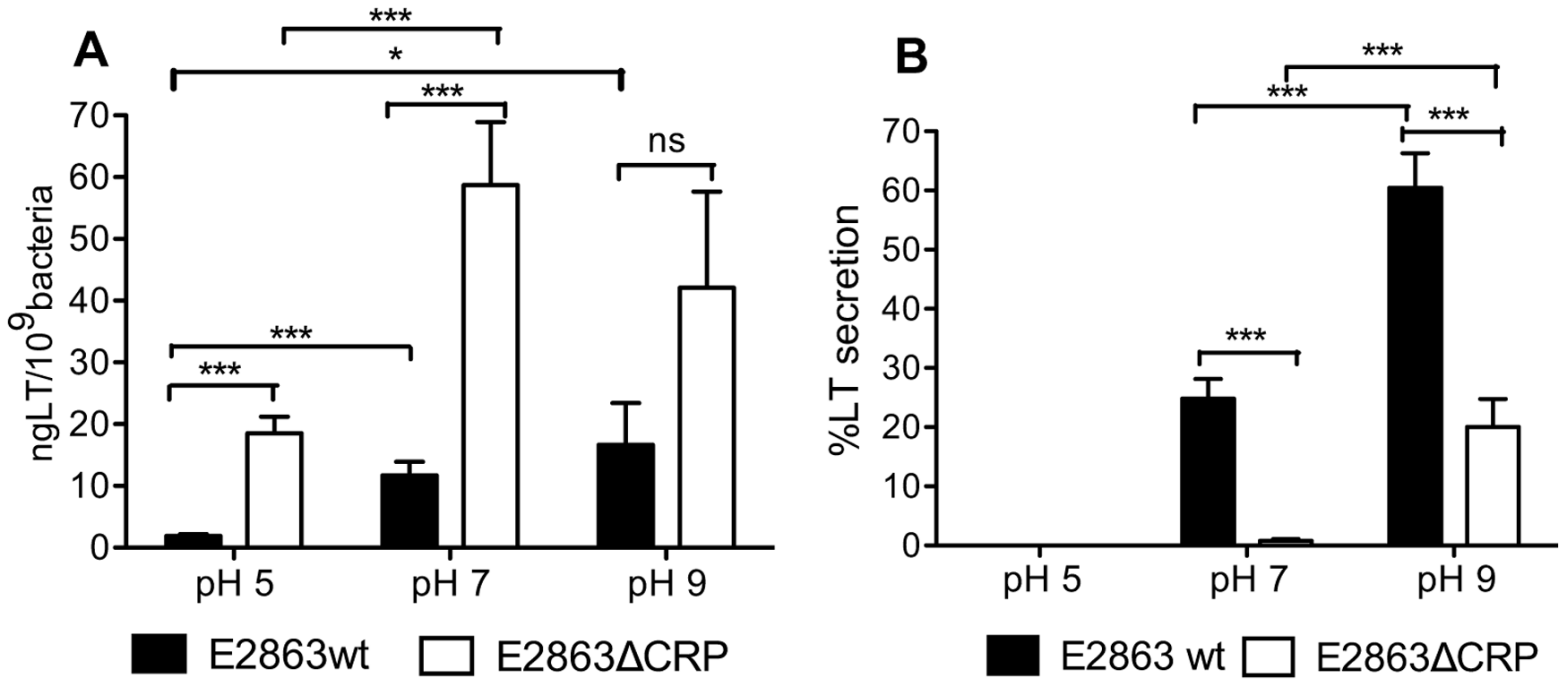
Effect of the pH on the total production (A) and secretion (B) of LT toxin. The wild-type (E2863 wt) and cAMP receptor protein deficient strain (E2863ΔCRP) were grown in LBK media adjusted to different pH values as described in Material and Methods. The concentration of LT toxin was determined by GM1-ELISA in the pellet and supernatant. Total production was defined as the total LT present in the pellet and supernatant and percentage of secretion as the ratio of LT in the supernatant to total LT. Bars represent the mean and error bars represent the standard deviations of 9 independent cultures. A Student t test was used to calculate P values, *P<0.05, **P<0.01, ***p<0.001.

In Luria Bertani broth (LB) cultures, which induce secretion of LT the pH of the medium increases to 8.5 or 9 when the culture reaches stationary phase. Growth in the human intestine also exposes infecting ETEC to alkaline pH in the duodenum and close to the epithelium. Hence, we were interested to analyse the effect of CRP under different pH conditions. We first established the effects of pH on the standard growth curve of E2863wt and E2863ΔCRP strains in LBK media buffered to pH 5, 7 and 9. Production and secretion of LT toxin were measured by ELISA and the number of *eltAB* transcripts was quantified by q-PCR at 3 and 5 hours and in overnight cultures. Total LT production at pH 5 and pH 7 was similar to production in unbuffered LBK media where pH increases gradually from pH 7.4 to 8.5 during growth. Again, LT production was higher in E2863ΔCRP than E2863wt at both pH 5 (p<0.001) and pH 7 (p<0.001). However no significant difference was found at pH 9 (Figure 3A) although a trend of higher LT production was observed in E2863ΔCRP compared to E2863wt. LT secretion was completely inhibited at pH 5 for both strains. At pH 7 and pH 9, secretion of LT was higher for E2863wt compared to E2863ΔCRP (p<0.001 and p<0.001, respectively) confirming that CRP is a positive regulator of secretion at neutral and alkaline pH. However, we observed that LT production and secretion in the wild-type is pH dependent and increases with pH. Indeed, the level of secretion at pH 9 reached very high values of around 60% of the produced LT compared to approximately 10–20% % under growth in unbuffered media and at pH 7 (compare Figure 1D and Figure 3B). Analysis of the effect of time in the production and secretion of LT toxin showed that LT production is higher at 3 hours compared to 5 hours and overnight for both pH 5 (p<0.001 and p<0.001, respectively) and pH 7 (p = 0.0352 and p = 0.0025, respectively), whereas secretion levels were similar during the time for pH 7 and pH 9 (data not shown). Thus, our data suggest that at pH 9 LT secretion is still positively regulated by CRP, but growth at pH 9 also further promotes both production and secretion resulting in maximal LT production and secretion in the wild type at pH 9.

### CRP is a Transcriptional Repressor of the eltAB Operon Independent of pH

**Figure 4:**
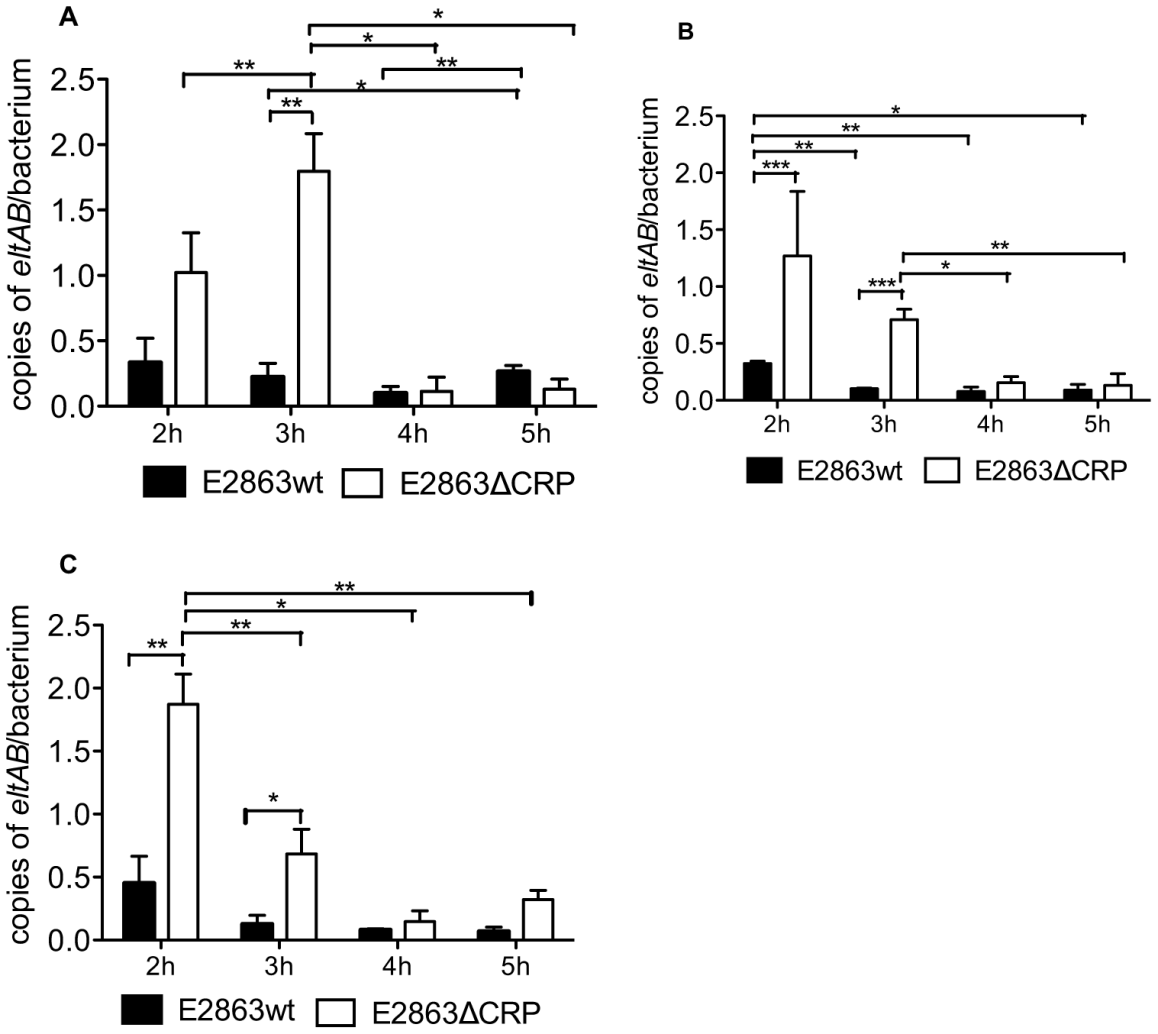
Transcription of the *eltAB* operon is repressed by CRP independently of pH. The gene expression levels were determined for E2863wt and E2863ΔCRP by q-PCR as transcripts per bacteria calculated from OD values of the cultures in LBK media adjusted at different pH. A) pH 5, B) pH 7 and C) pH 9. The bars represent the mean value of 4 analyses. A Student t test was used to calculate the P values, *P<0.05, **P<0.01, ***p<0.001.

The pH dependency observed for the LT protein could depend on posttranscriptional effects such as assembly of the LT holotoxin in the periplasm. To determine the transcriptional regulation we performed q-PCR analyses on transcription after 2, 3, 4 and 5 hours in the different pH conditions. The results showed that *eltAB* operon is repressed by CRP independent of pH. Higher numbers of *eltAB* transcripts were found in E2863ΔCRP compared to E2863wt at all pH ranges. At pH 5 repression was found at 3 hours (p = 0.0022) (Figure 4A), whereas at pH 7 CRP repression was present at 2 hours and 3 hours (p<0.0001 and p<0.0001, respectively) (Figure 4B) and in pH 9 at 2 hours and 3 hours (p = 0.0046 and p = 0.0373, respectively) (Figure 4C). We also observed a higher expression of both E2863wt and E2863ΔCRP in the early hours of growth and a significant decreased of number of *eltAB* transcripts over time irrespective of pH. The levels of the E2863ΔCRP were significantly decreased after 4 and 5 hours with respect to 2 and 3 hours (Figure 4A–C). The results suggest that repression of *eltAB* by CRP does not depend on pH. We compared the expression of *eltAB* after 2 and 3 hours in E2863wt grown at pH 5, 7 and 9 and no significant increase in expression levels were observed at pH 9. Hence the increased levels of assembled LT toxin at pH 9 compared to pH 5 is probably due to post-transcriptional events.

### Intracellular Levels of cAMP are Increased in a CRP Mutant

**Figure 5:**
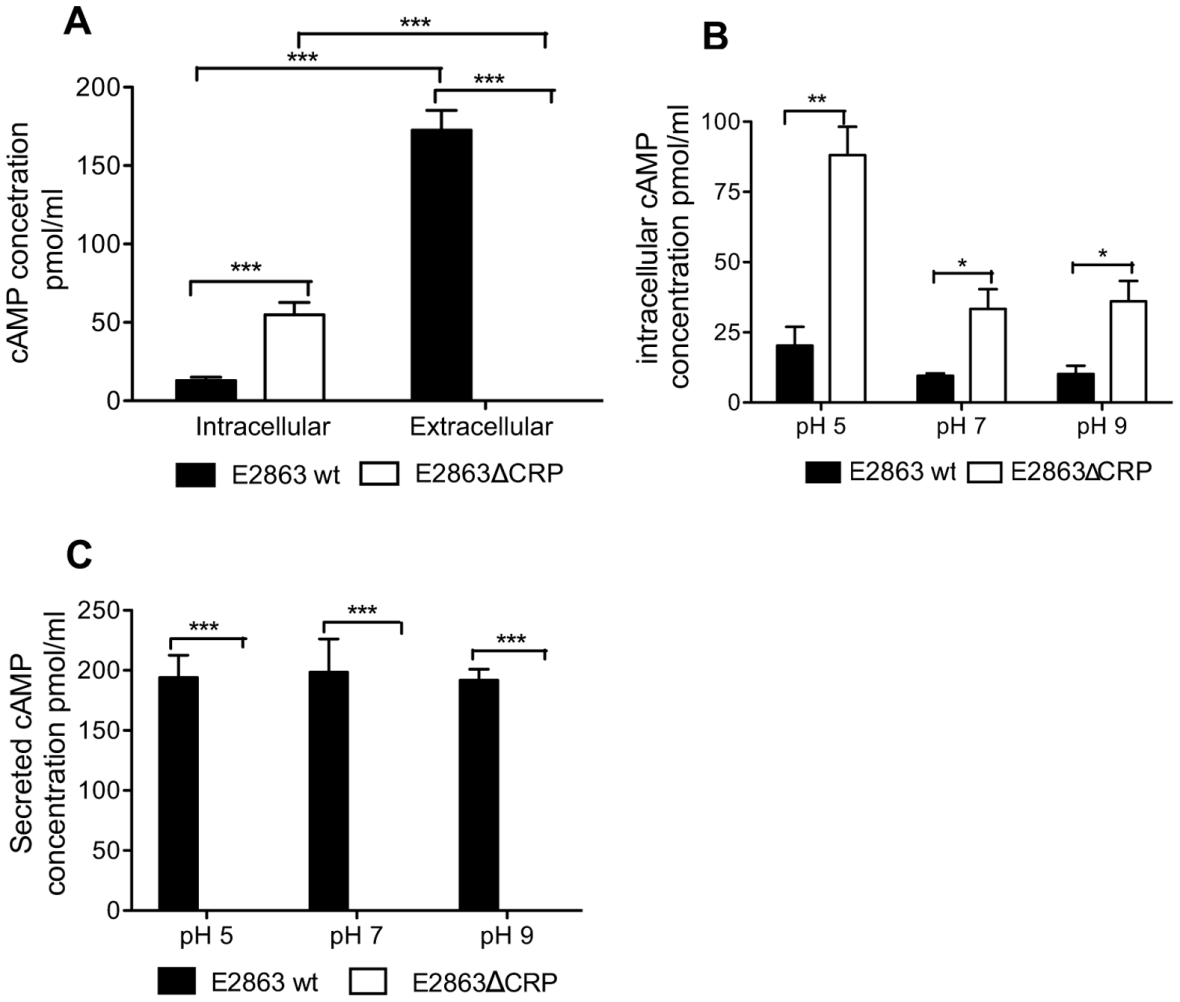
Intracellular levels of cAMP are increased in a CRP mutant. cAMP was measured by cAMP ELISA assay in the pellet and supernatant in E2863wt and E2863ΔCRP strains. The strains were grown in LBK media (A) and in LBK media adjusted at different pH (B and C) for 3 hours. Intracellular cAMP is defined as the cAMP detected in the pellet and extracellular cAMP as the cAMP present in the supernatant. Bars represent the mean and error bars represent the standard deviations of 4 independent experiments. A Student t test was used to calculate P values *P<0.05, **P<0.01, ***p<0.001. A) cAMP in LBK media, B) intracellular cAMP at different pH ranges, C) extracellular cAMP at different pH ranges.

We were interested in why the pH effect on secretion of LT might occur and performed a number of experiments to address this question. Firstly, the secretion of LT was positively regulated by CRP and increased with increasing pH. cAMP binds to the cyclic receptor protein CRP and forms the active cAMP-CRP complex that affects the expression of more than 200 genes in *E. coli*. An effect of pH on intracellular levels of cAMP has previously been reported [34]. We hypothesized that alkaline pH might increase the intracellular levels of cAMP at pH 9 which could account for the increased cAMP-CRP mediated secretion. In order to investigate if the levels of cAMP were affected in the wild-type and mutant strain, and if it was pH dependent, the levels of cAMP were measured by ELISA in the pellet and supernatant of the E2863wt and E2863ΔCRP strains growth in unbuffered LBK media and in media adjusted to different pH. The intracellular cAMP levels were found to be higher in the E2863ΔCRP compared to E2863wt (p<0.001), but the extracellular cAMP levels were higher in the E2863wt compared to E2863ΔCRP (p<0.001) since 75% of the produced cAMP was found in the supernatant (Figure 5A). The total level of cAMP was higher in the E2863wt indicating that CRP directly or indirectly promotes production of cAMP. The mutant was completely devoid of secretion of cAMP into the medium, which might indicate that the E2863ΔCRP is defective in general in transport of substances into the surrounding medium. At all the different pH values the levels of cAMP were higher extracellular for the E2863wt and higher intracellular for the E2863ΔCRP but differences between pH was not observed in this study (Figures 5B and 5C). We conclude that intracellular levels of cAMP are higher in the E2863ΔCRP, which might depend on defective secretion of excess cAMP since the E2863wt secreted 75% of its produced cAMP. However cAMP production is pH independent and altered levels of cAMP cannot explain the secretion effect at pH 9.

### The Effect of pH on the Bacteria Affect Growth but not Viability

To colonize the human gastrointestinal tract bacteria must be able to grow at different pH conditions until it can reach the site of infection. However, growth rate and viability of bacteria can be affected under different conditions, and it is possible that the increased secretion at pH 9 could be due to membrane leakage as alkaline pH poses a stress on *E. coli* membranes. Hence, the viability and membrane integrity was analyzed. We registered the optical density at 3, 5 hours and from overnight cultures, and determined the viability of bacteria grown at different pH values using the Live-Dead viability kit which enables to detect bacteria with intact cell membranes (live) and bacteria with damaged membrane (dead) by flow cytometer in the wild-type E2863wt strain and the mutant strain E2863ΔCRP. The wild-type E2863wt strain had a better growth compared to E2863ΔCRP in all pH conditions. No difference was observed between pH 7 and LBK media. However a difference was observed between pH values. E2863wt grown at pH 5 grew slower than during growth at pH 7 in the exponential phase but reached the highest OD value at the stationary phase (OD_600_ 6.13±0.48) compared to pH 7 (OD_600_ 5.52±0.61) and pH 9 (OD_600_ 4.73±1.81). In contrast, growth at pH 9 was slow at all points of the growth curve (Table S1). Analysis of the viability of bacteria showed that there were no significant differences of the percentage of live bacteria compared to dead bacteria at the different pH conditions for both strains. However, at pH 9 compromised bacteria, which had started to take up some of the dye were more frequent, suggesting that at pH 9 there is a change of permeability of the membrane compared to bacteria growth at pH 5 and pH 7 (Table S1). We conclude that the highest OD levels after overnight growth of the E2863wt occurs at pH 5 while growth was reduced at pH 9, suggesting that bacteria can multiply at high rates in an acidic pH environment like the human stomach and that growth rate decreases when ETEC reaches a basic environment such as parts of the small intestine. However both E2863wt and E2863ΔCRP maintained viability at all pH and hence the pH effect on secretion is not dependent on leaky membranes.

### Secretion of LT is Regulated by CRP in vivo

**Figure 6:**
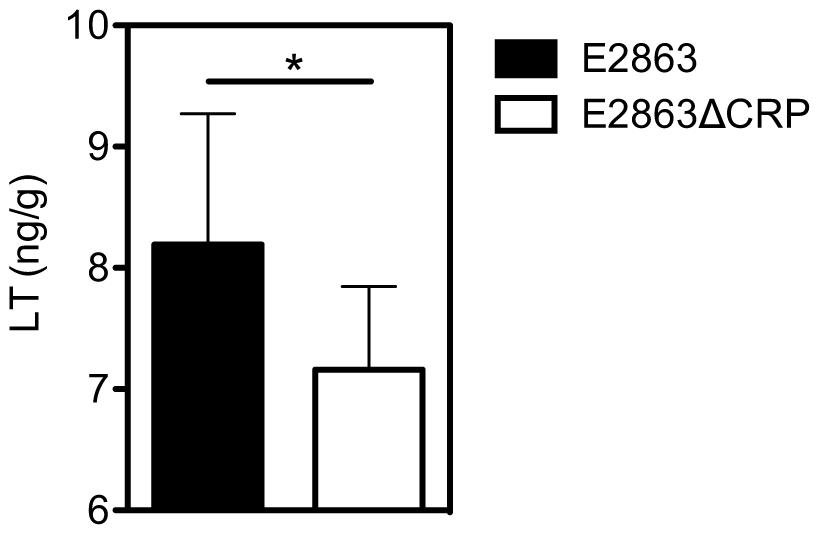
CRP is a positive regulator of LT secretion *in vivo*. The wild-type (E2863 wt) and cAMP receptor protein deficient strain E2863ΔCRP) were appropriately diluted in PBS and orogastrically inoculated into infant mice. Three hours post-infection 8 small intestines per bacterial strain were collected and used for estimation of LT secretion by GM1-ELISA as described in Material and Methods. Bars represent the geometric mean and error bars represent the standard deviations of 3 independent experiments. A Student t test was used to calculate P values, *P<0.05.

Our results indicate that the conditions in the small intestine might favor LT secretion. In order to verify our results *in vivo,* we used an infant mice model of 3 days old pups to determine small intestinal colonization and LT secretion *in vivo*. Using a competition assay previously used for *V. cholerae* [31] where the mice were fed equal amounts of E2863ΔCRP and E2863wt, we first established that E2863ΔCRP is defective in colonization relative to E2863wt. This was expected since the mutant was found to be immobile and unable to form biofilm (data not shown). In further experiments we divided the mice into groups and fed the E2863ΔCRP and E2863wt strains individually. The mice were sacrificed after 3 and 18 hours and the small intestines were weighed and used to estimate either pH and CFUs or *in vivo* LT secretion. The pH of the duodenum and ileum was measured and although 7 day old mice in earlier studies were found to have developed a pH gradient where duodenum registered a pH of 6 to 7, and the ileum presented a higher pH of 8, the 3 day old mice had no measurable pH gradient. In agreement with the results from the competition experiment, the geometric mean small intestinal colonization of 3 day old pups were found to be lower by the CRP mutant at both 3 and 18 hours post-infection as compared with the WT bacteria, however these estimates were not significantly different (data not shown). No significant difference in colonization measured as CFU was detected after 3 and 18 hours or in the upper and lower part of the intestine of the 3 day old pups. The levels of secreted LT were measured in the supernatant after centrifugation of the intestines. Interestingly, the levels of secreted LT measured in the small intestinal luminal supernatant after centrifugation was significantly higher 3 hours post-infection with E2863wt compared to E2863ΔCRP (p = 0.04) (Figure 6). Surprisingly low levels of secreted LT were detected after 18 hours in the small intestines of both groups (data not shown). These results confirmed that LT is expressed and secreted at early time points also *in vivo* and that CRP is a positive regulator of secretion also *in vivo*.

## Discussion

**Figure 7:**
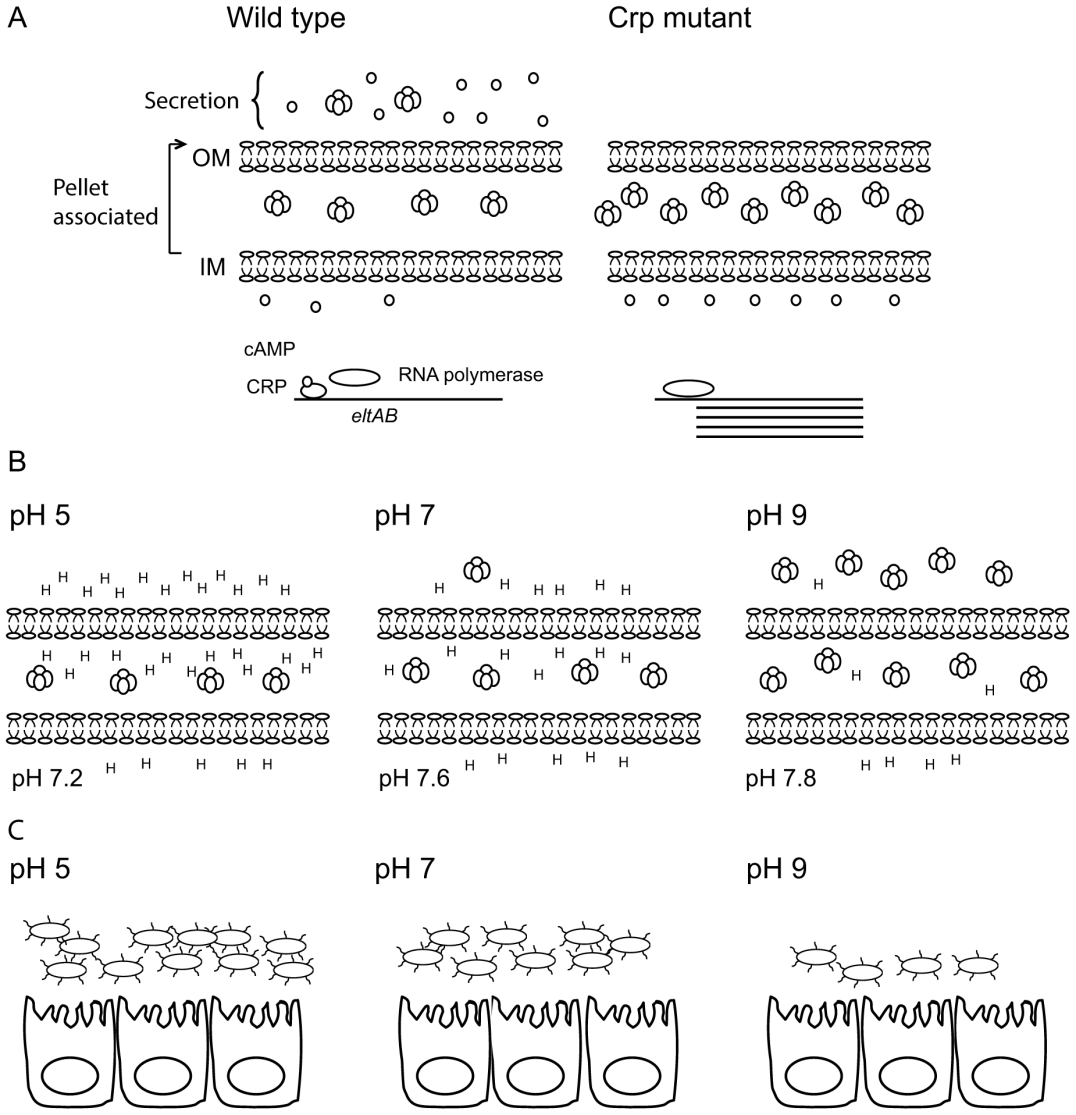
Model of total LT production and secretion. A) The total production defined at LT both in the pellet fraction and in the supernatant fraction was higher in E2863ΔCRP than in the wild-type E2863 confirming that transcription of the *eltAB* operon is induced in the absence of cAMP-CRP. Secretion was decreased in E2863ΔCRP demonstrating that CRP is a positive regulator of LT secretion. B) At pH 5 secretion of LT is inhibited and the outer environment and the periplasmic space is acidic and the proton gradient favors an influx of protons into the cytosol. In order to keep the cytosol close to neutral there is a net outflux of protons from the cytosol. At neutral pH the cytosol is kept slightly more alkaline than the periplasm favouring a normal proton gradient and some LT is secreted At pH 9 the proton concentration is lower in the periplasm than the cytosol creating an inverted proton gradient and LT production and secretion is induced in response to the alkaline conditions in the periplasm. C) The inverted proton gradient poses a stress for the bacterial cell resulting in lower growth rate at pH 9 while growth at pH 5 supported growth to higher OD values of bacterial cells than pH 9.

One of the most important virulence factors of wild type strains of ETEC is the production and release of LT toxin. In contrast to *Vibrio cholerae* that secrete the homologous cholera toxin CT, ETEC strains are not proficient at secreting LT into the extracellular media [35] and are instead suggested to keep the majority of the toxin produced attached to the membranes or in the periplasm [36], [37]. However, some strains have been reported to secrete up to 50% of the produced toxin [14]. To study the regulation of the total LT production as well as the secretion of LT toxin, we measured the total production of LT which represents the quantity of LT detected in the pellet and the supernatant of the cultured bacteria e.g. toxin inside the bacteria and the one that has been released (Figure 7A). We measured the percentage of secretion representing the toxin that has been released and divided it by the total amount of toxin. In addition, we studied the role of the CRP regulator in the production and secretion of LT toxin by the construction of CRP mutants. We found that CRP negatively regulates the production of LT as a repressor of the *eltAB* gene. These findings are in agreement with earlier analyses of H10407 that demonstrated that the cAMP-CRP complex represses transcription of heat-labile toxin genes [16]. Our results contradict one earlier study that reported that cAMP-CRP is a positive regulator of the *eltAB* operon [18]. However, a later study has suggested that there might be inter-strain differences in transcription of essential virulence genes in response to added glucose [38]. To validate our findings, we performed the analysis of the role of CRP in two different strains, E2863 (LT, CS6) and as well as H10407 (LT, STh, STp, CFA/I) and we also followed the effect of CRP in exponential and stationary growth phase in E2863wt and ΔCRP. We found the same pattern of the role of CRP in both strains confirming that CRP is a negative regulator of the transcription of *eltAB* in the strains used in this study (Figure 7A).

Our results further showed that CRP is a positive regulator of the secretion of the heat-labile toxin. This constitutes the first report that associates a role of CRP as a positive regulator of secretion of LT and provides an explanation to previous conflicting results on the role of glucose and/or CRP on measured amounts of LT. Addition of glucose has been reported to increase LT activity and yield [16], [36], [39]. Glucose would lower the cAMP-CRP dependent repression of the *eltAB* promoter and result in higher levels of LT, however our results show that at the same time secretion is repressed in the presence of glucose. The contradictory results from different studies depend on whether the secreted or total production is measured in the assay. Kunkel and Robertson state that the increase in LT activity they report is probably dependent on increased production and not secretion [36]. Other studies have added polymyxin or linkomycin to the cultures before analyzing for toxin secretion, which would release periplasmic LT into the media [16], [39]. Our results show that CRP has two independent regulatory roles in LT production and secretion and is a positive regulator of secretion. From a biological point of view the lower parts of the small intestine where ETEC colonizes is probably glucose limited and in addition a variant of the other major toxin STh has been shown to be positively regulated by CRP [16]. A hypothesis that the two toxins ST and LT are released at different parts of the intestine based on the glucose/CRP regulation has previously been proposed by us and by Bodero and Munson [16], [40] but the new results presented in the current study favour the hypothesis that the release of toxins are co-regulated since high levels of cAMP would favour secretion of both ST and LT.

We found that the production of the heat-labile toxin is CRP-dependent at pH 5 and pH 7 but not significantly down-regulated by CRP at pH 9. However we observed that this effect was not present at the transcriptional level were regulation of *eltAB* transcripts was shown to be pH independent. A pH dependency of LT secretion/activity was observed early and has been reported repeatedly [17], [36], [39], [41], [42]. Since growth on glucose acidifies the media and growth in minimal media does not increase pH to the same degree as in LB media, several studies using glucose supplemented media observed the need to adjust the pH in order to detect LT activity [17], [36]. We show here that heat-labile secretion was completely absent at pH 5 and increased significantly towards neutral and basic pH. The highest secretion values were found after growth in media buffered to pH 9 where E2863wt, which normally secrete approximately 10% of its produced LT were able to secrete up to 60%. Hence, both alkaline pH and CRP are positive regulators of the level of secretion.

The disulfide bridge protein DsbA has been implicated in assembly of the LT holotoxin by forming disulfide bonds between the B subunits [10], [11], [43] while DegP removed misfolded proteins [10]. Both DsbA and DegP have been reported to be induced by alkaline pH [44], [45]. Spontaneous assembly of the B subunits has also been shown and recently it was shown that spontaneous assembly is pH dependent and increase with increasing pH, no assembly was detected below pH 7 [8]. Since the periplasmic space has similar pH as the surrounding environment [22] this could provide an explanation to the pH dependence on LT production and assembly shown in this study. However, although we speculate that the periplasmic pH is important for LT assembly in the periplasm it does not explain the higher levels of LT secretion and LT was indeed detected found in the periplasm at pH 5 indicating that LT assembly at acidic pH is occurring. In *Vibrio cholerae* that secrete the homologous cholera toxin CT a dependence on alkaline pH for secretion has also been reported [46]. CT was shown to be localized close to the outer membrane in response to alkaline pH which favoured secretion [46]. Due to the similarities of CT and LT we believe that similar mechanisms are present in ETEC.

The total levels of [cAMP] were higher in E2863wt than E2863ΔCRP, which would suggest that CRP directly or indirectly is a positive regulator of cAMP production. However, cAMP is generated by adenylate cyclase (AC) encoded by *cya* in response to phosphorylated EIIA^Glc^ and transcription of *cya* has been reported to be negatively regulated by CRP [47]. In addition degradation of cAMP by phosphodiesterase CpdA is positively regulated by CRP to maintain homeostatic levels of cAMP. An interesting result was that we found significantly higher levels of extracellular cAMP in the wild-type strain. It has been previously reported that the levels of intracellular cAMP in the bacteria need to be controlled in order for *E. coli* to be able to respond to changes in carbon sources and to form the cAMP-CRP complex [48]. *In vitro* studies have shown that *E. coli* seem to mainly excrete surplus cAMP to maintain its intracellular levels of cAMP at physiological concentrations. Up to 90% of the produced cAMP might be found in the supernatant [49]. As mentioned cAMP is degraded by the phosphodiesterase CpdA which is indirectly activated by the cAMP-CRP complex to maintain homeostatic levels of cAMP [50]. This export has been shown to be dependent on an active energy-dependent mechanism that is dependent on TolC [51], [52]. TolC is a cytoplasmic membrane protein involved in export of toxic metabolites such as antibiotics. TolC is also involved in secretion of the heat-stable toxin ST [53], [54]. The expression of TolC is positively regulated by the MarRAB operon that in turn is induced by crp-cAMP. Our study has also shown a depletion of the secretion system in the CRP mutant strain and therefore higher level of intracellular cAMP compared to the wild-type strain suggesting that the absence of extracellular cAMP and the increased intracellular cAMP concentrations in the CRP mutant might mainly be dependent of reduced expression of TolC. The *tolC* locus is located close to and in opposite direction to the *MutT, ygiB cpdA ygiA* operon in and the latter operon was shown to be positively regulated by CRP-cAMP in *Vibrio vulnificus* [55]. Hence, based on our findings we propose that the cAMP phosphodiesterase might be reduced in the ETEC CRP mutant thereby reducing the degradation of formed cAMP. In conclusion, the impaired export of cAMP and the reduced degradation of cAMP may account for the higher levels of intracellular cAMP found in a CRP mutant but the higher total levels of cAMP produced by the wildtype is not explained by this and regulation of cAMP in CRP mutants needs to be further investigated.

In summary we propose that ETEC use the pH gradient in the gastrointestinal tract to sense when it has reached the small intestine and colonize its appropriate niche. In fact alkaline surface microclimates in the small intestine have been described [56]. The pH is expected to increase during entry into the duodenum, in the distal small intestine and close to the epithelium, while entry into the large bowels probably results in a drop in pH. In agreement with this H10407 was found to colonize the ileum in the distal part of adult mice where pH is more alkaline [57] and we found a tendency of higher colonization in the lower parts of the intestine in 7 day old mice (data not shown). We also show for the first time that CRP is a positive regulator of LT secretion and that external pH and CRP together regulate induction of transcription and maximal release of LT at alkaline pH.

## Supporting Information

Table S1 (DOCX)

Table S2 (DOCX)

## References

[1] Bryce J, Boschi-Pinto C, Shibuya K, Black RE (2005) WHO estimates of the causes of death in children. Lancet 365: 1147–1152. 15794969 10.1016/S0140-6736(05)71877-8

[2] Qadri F, Svennerholm AM, Faruque AS, Sack RB (2005) Enterotoxigenic Escherichia coli in developing countries: epidemiology, microbiology, clinical features, treatment, and prevention. Clin Microbiol Rev 18: 465–483. 16020685 10.1128/CMR.18.3.465-483.2005 PMC1195967

[3] Wenneras C, Erling V (2004) Prevalence of enterotoxigenic Escherichia coli-associated diarrhoea and carrier state in the developing world. J Health Popul Nutr 22: 370–382. 15663170

[4] Moss J, Richardson SH (1978) Activation of adenylate cyclase by heat-labile Escherichia coli enterotoxin. Evidence for ADP-ribosyltransferase activity similar to that of choleragen. J Clin Invest 62: 281–285. 209060 10.1172/JCI109127 PMC371764

[5] Spangler BD (1992) Structure and function of cholera toxin and the related Escherichia coli heat-labile enterotoxin. Microbiol Rev 56: 622–647. 1480112 10.1128/mr.56.4.622-647.1992 PMC372891

[6] Nataro JP, Kaper JB (1998) Diarrheagenic Escherichia coli. Clin Microbiol Rev 11: 142–201. 9457432 10.1128/cmr.11.1.142 PMC121379

[7] Hofstra H, Witholt B (1985) Heat-labile enterotoxin in Escherichia coli. Kinetics of association of subunits into periplasmic holotoxin. J Biol Chem 260: 16037–16044. 3905802

[8] Zrimi J, Ng Ling A, Giri-Rachman Arifin E, Feverati G, Lesieur C (2010) Cholera toxin B subunits assemble into pentamers–proposition of a fly-casting mechanism. PLoS One 5: e15347. 21203571 10.1371/journal.pone.0015347 PMC3006222

[9] Findlay G, Hirst TR (1994) Escherichia coli and Vibrio cholerae strains deficient in an enzyme involved in disulphide bond formation (DsbA) show an increase in sensitivity to dithiothreitol. Biochem Soc Trans 22: 77S. 8206309 10.1042/bst022077s

[10] Wulfing C, Rappuoli R (1997) Efficient production of heat-labile enterotoxin mutant proteins by overexpression of dsbA in a degP-deficient Escherichia coli strain. Arch Microbiol 167: 280–283. 9094224 10.1007/s002030050444

[11] Yu J, Webb H, Hirst TR (1992) A homologue of the Escherichia coli DsbA protein involved in disulphide bond formation is required for enterotoxin biogenesis in Vibrio cholerae. Mol Microbiol 6: 1949–1958. 1324389 10.1111/j.1365-2958.1992.tb01368.x

[12] Horstman AL, Kuehn MJ (2000) Enterotoxigenic Escherichia coli secretes active heat-labile enterotoxin via outer membrane vesicles. J Biol Chem 275: 12489–12496. 10777535 10.1074/jbc.275.17.12489 PMC4347834

[13] Horstman AL, Kuehn MJ (2002) Bacterial surface association of heat-labile enterotoxin through lipopolysaccharide after secretion via the general secretory pathway. J Biol Chem 277: 32538–32545. 12087095 10.1074/jbc.M203740200 PMC4391702

[14] Lasaro MA, Rodrigues JF, Mathias-Santos C, Guth BE, Regua-Mangia A, et al (2006) Production and release of heat-labile toxin by wild-type human-derived enterotoxigenic Escherichia coli. FEMS Immunol Med Microbiol 48: 123–131. 16965360 10.1111/j.1574-695X.2006.00134.x

[15] Wai SN, Takade A, Amako K (1995) The release of outer membrane vesicles from the strains of enterotoxigenic Escherichia coli. Microbiol Immunol 39: 451–456. 8569529 10.1111/j.1348-0421.1995.tb02228.x

[16] Bodero MD, Munson GP (2009) Cyclic AMP receptor protein-dependent repression of heat-labile enterotoxin. Infect Immun 77: 791–798. 19075028 10.1128/IAI.00928-08 PMC2632052

[17] Gilligan PH, Robertson DC (1979) Nutritional requirements for synthesis of heat-labile enterotoxin by enterotoxigenic strains of Escherichia coli. Infect Immun 23: 99–107. 33900 10.1128/iai.23.1.99-107.1979 PMC550695

[18] Gibert I, Villegas V, Barbe J (1990) Expression of Heat-labile Enterotoxin Genes is under Cyclic AMP Control in Escherichia coli. Current Microbiology 20: 83–90.

[19] de Jonge R, Takumi K, Ritmeester WS, van Leusden FM (2003) The adaptive response of Escherichia coli O157 in an environment with changing pH. J Appl Microbiol 94: 555–560. 12631190 10.1046/j.1365-2672.2003.01865.x

[20] Slonczewski JL, Rosen BP, Alger JR, Macnab RM (1981) pH homeostasis in Escherichia coli: measurement by 31P nuclear magnetic resonance of methylphosphonate and phosphate. Proc Natl Acad Sci U S A 78: 6271–6275. 7031646 10.1073/pnas.78.10.6271 PMC349020

[21] Zilberstein D, Agmon V, Schuldiner S, Padan E (1984) Escherichia coli intracellular pH, membrane potential, and cell growth. J Bacteriol 158: 246–252. 6325389 10.1128/jb.158.1.246-252.1984 PMC215405

[22] Wilks JC, Slonczewski JL (2007) pH of the cytoplasm and periplasm of Escherichia coli: rapid measurement by green fluorescent protein fluorimetry. J Bacteriol 189: 5601–5607. 17545292 10.1128/JB.00615-07 PMC1951819

[23] Skorupski K, Taylor RK (1996) Positive selection vectors for allelic exchange. Gene 169: 47–52. 8635748 10.1016/0378-1119(95)00793-8

[24] Vaitkevicius K, Lindmark B, Ou G, Song T, Toma C, et al (2006) A Vibrio cholerae protease needed for killing of Caenorhabditis elegans has a role in protection from natural predator grazing. Proc Natl Acad Sci U S A 103: 9280–9285. 16754867 10.1073/pnas.0601754103 PMC1482601

[25] Valeru SP, Rompikuntal PK, Ishikawa T, Vaitkevicius K, Sjoling A, et al (2009) Role of melanin pigment in expression of Vibrio cholerae virulence factors. Infect Immun 77: 935–942. 19103773 10.1128/IAI.00929-08 PMC2643646

[26] Datsenko KA, Wanner BL (2000) One-step inactivation of chromosomal genes in Escherichia coli K-12 using PCR products. Proc Natl Acad Sci U S A 97: 6640–6645. 10829079 10.1073/pnas.120163297 PMC18686

[27] Zhao G, Zhu L, Feng E, Cao X, Shang N, et al (2010) A novel anti-virulence gene revealed by proteomic analysis in Shigella flexneri 2a. Proteome Sci 8: 30. 20540790 10.1186/1477-5956-8-30 PMC2904734

[28] Sjoling A, Wiklund G, Savarino SJ, Cohen DI, Svennerholm AM (2007) Comparative analyses of phenotypic and genotypic methods for detection of enterotoxigenic Escherichia coli toxins and colonization factors. J Clin Microbiol 45: 3295–3301. 17687011 10.1128/JCM.00471-07 PMC2045327

[29] Lothigius A, Janzon A, Begum Y, Sjoling A, Qadri F, et al (2008) Enterotoxigenic Escherichia coli is detectable in water samples from an endemic area by real-time PCR. J Appl Microbiol 104: 1128–1136. 17976169 10.1111/j.1365-2672.2007.03628.x

[30] Sjoling A, Qadri F, Nicklasson M, Begum YA, Wiklund G, et al (2006) In vivo expression of the heat stable (estA) and heat labile (eltB) toxin genes of enterotoxigenic Escherichia coli (ETEC). Microbes Infect 8: 2797–2802. 17045506 10.1016/j.micinf.2006.08.011

[31] Nygren E, Li BL, Holmgren J, Attridge SR (2009) Establishment of an adult mouse model for direct evaluation of the efficacy of vaccines against Vibrio cholerae. Infect Immun 77: 3475–3484. 19470748 10.1128/IAI.01197-08 PMC2715679

[32] Nilsson G, Belasco JG, Cohen SN, von Gabain A (1984) Growth-rate dependent regulation of mRNA stability in Escherichia coli. Nature 312: 75–77. 6387508 10.1038/312075a0

[33] Battesti A, Majdalani N, Gottesman S (2011) The RpoS-mediated general stress response in Escherichia coli. Annu Rev Microbiol 65: 189–213. 21639793 10.1146/annurev-micro-090110-102946 PMC7356644

[34] Ma Z, Richard H, Foster JW (2003) pH-Dependent modulation of cyclic AMP levels and GadW-dependent repression of RpoS affect synthesis of the GadX regulator and Escherichia coli acid resistance. J Bacteriol 185: 6852–6859. 14617649 10.1128/JB.185.23.6852-6859.2003 PMC262709

[35] Clements JD, Lowe KL, Bonham L, el-Morshidy S (1985) Intracellular distribution of heat-labile enterotoxin in a clinical isolate of Escherichia coli. Infect Immun 50: 317–319. 3899935 10.1128/iai.50.1.317-319.1985 PMC262174

[36] Kunkel SL, Robertson DC (1979) Factors affecting release of heat-labile enterotoxin by enterotoxigenic Escherichia coli. Infect Immun 23: 652–659. 37162 10.1128/iai.23.3.652-659.1979 PMC414214

[37] Wensink J, Gankema H, Jansen WH, Guinee PA, Witholt B (1978) Isolation of the membranes of an enterotoxigenic strain of Escherichia coli and distribution of enterotoxin activity in different subcellular fractions. Biochim Biophys Acta 514: 128–136. 214115 10.1016/0005-2736(78)90082-2

[38] Kansal R, Rasko DA, Sahl JW, Munson GP, Roy K, et al (2013) Transcriptional Modulation of Enterotoxigenic Escherichia coli Virulence Genes in Response to Epithelial Cell Interactions. Infect Immun 81: 259–270. 23115039 10.1128/IAI.00919-12 PMC3536156

[39] Hengge R (2009) Principles of c-di-GMP signalling in bacteria. Nat Rev Microbiol 7: 263–273. 19287449 10.1038/nrmicro2109

[40] Nicklasson M, Sjoling A, Lebens M, Tobias J, Janzon A, et al (2008) Mutations in the periplasmic chaperone leading to loss of surface expression of the colonization factor CS6 in enterotoxigenic Escherichia coli (ETEC) clinical isolates. Microb Pathog 44: 246–254. 18037262 10.1016/j.micpath.2007.06.009

[41] Minami J, Okabe A, Nagata A, Hayashi H (1984) Quantitative analysis of the production of heat-labile enterotoxin by enterotoxigenic Escherichia coli. Acta Med Okayama 38: 461–469. 6393717 10.18926/AMO/30338

[42] Mundell DH, Anselmo CR, Wishnow RM (1976) Factors influencing heat-labile Escherichia coli enterotoxin activity. Infect Immun 14: 383–388. 9363 10.1128/iai.14.2.383-388.1976 PMC420895

[43] Hirst TR, Nashar TO, Eaglestone S, Lencer WI, Webb HM, et al (1994) Bacterial and host interactions during the biogenesis, toxicity and immunogenicity of Escherichia coli heat-labile enterotoxin. Biochem Soc Trans 22: 306–309. 7958313 10.1042/bst0220306

[44] Malhotra S, Silo-Suh LA, Mathee K, Ohman DE (2000) Proteome analysis of the effect of mucoid conversion on global protein expression in Pseudomonas aeruginosa strain PAO1 shows induction of the disulfide bond isomerase, dsbA. J Bacteriol 182: 6999–7006. 11092861 10.1128/jb.182.24.6999-7006.2000 PMC94826

[45] Thede GL, Arthur DC, Edwards RA, Buelow DR, Wong JL, et al (2011) Structure of the periplasmic stress response protein CpxP. J Bacteriol 193: 2149–2157. 21317318 10.1128/JB.01296-10 PMC3133086

[46] Aoki H, Wu H, Nakano T, Ooi Y, Daikoku E, et al (2009) Nanotransportation system for cholera toxin in Vibrio cholerae 01. Med Mol Morphol 42: 40–46. 19294491 10.1007/s00795-008-0431-x

[47] Aiba H (1985) Transcription of the Escherichia coli adenylate cyclase gene is negatively regulated by cAMP-cAMP receptor protein. J Biol Chem 260: 3063–3070. 2982847

[48] Gosset G, Zhang Z, Nayyar S, Cuevas WA, Saier MH Jr (2004) Transcriptome analysis of Crp-dependent catabolite control of gene expression in Escherichia coli. J Bacteriol 186: 3516–3524. 15150239 10.1128/JB.186.11.3516-3524.2004 PMC415760

[49] Bettenbrock K, Sauter T, Jahreis K, Kremling A, Lengeler JW, et al (2007) Correlation between growth rates, EIIACrr phosphorylation, and intracellular cyclic AMP levels in Escherichia coli K-12. J Bacteriol 189: 6891–6900. 17675376 10.1128/JB.00819-07 PMC2045212

[50] Kimata K, Takahashi H, Inada T, Postma P, Aiba H (1997) cAMP receptor protein-cAMP plays a crucial role in glucose-lactose diauxie by activating the major glucose transporter gene in Escherichia coli. Proc Natl Acad Sci U S A 94: 12914–12919. 9371775 10.1073/pnas.94.24.12914 PMC24238

[51] Goldenbaum PE, Hall GA (1979) Transport of cyclic adenosine 3′,5′-monophosphate across Escherichia coli vesicle membranes. J Bacteriol 140: 459–467. 227841 10.1128/jb.140.2.459-467.1979 PMC216670

[52] Hantke K, Winkler K, Schultz JE (2011) Escherichia coli exports cyclic AMP via TolC. J Bacteriol 193: 1086–1089. 21183666 10.1128/JB.01399-10 PMC3067598

[53] Foreman DT, Martinez Y, Coombs G, Torres A, Kupersztoch YM (1995) TolC and DsbA are needed for the secretion of STB, a heat-stable enterotoxin of Escherichia coli. Mol Microbiol 18: 237–245. 8709843 10.1111/j.1365-2958.1995.mmi_18020237.x

[54] Yamanaka H, Nomura T, Fujii Y, Okamoto K (1998) Need for TolC, an Escherichia coli outer membrane protein, in the secretion of heat-stable enterotoxin I across the outer membrane. Microb Pathog 25: 111–120. 9790870 10.1006/mpat.1998.0211

[55] Kim HM, Xu Y, Lee M, Piao S, Sim SH, et al (2010) Functional relationships between the AcrA hairpin tip region and the TolC aperture tip region for the formation of the bacterial tripartite efflux pump AcrAB-TolC. J Bacteriol 192: 4498–4503. 20581201 10.1128/JB.00334-10 PMC2937362

[56] Mizumori M, Ham M, Guth PH, Engel E, Kaunitz JD, et al (2009) Intestinal alkaline phosphatase regulates protective surface microclimate pH in rat duodenum. J Physiol 587: 3651–3663. 19451200 10.1113/jphysiol.2009.172270 PMC2742288

[57] Allen KP, Randolph MM, Fleckenstein JM (2006) Importance of heat-labile enterotoxin in colonization of the adult mouse small intestine by human enterotoxigenic Escherichia coli strains. Infect Immun 74: 869–875. 16428729 10.1128/IAI.74.2.869-875.2006 PMC1360293

