# Supplementary material for: Alkaline pH Is a Signal for Optimal Production and Secretion of the Heat Labile Toxin, LT in Enterotoxigenic Escherichia Coli (ETEC)
Source: PLoS One. 2013 Sep 18;8(9):e74069. doi: 10.1371/journal.pone.0074069 (PMC3776858; doi:10.1371/journal.pone.0074069)
Supplement: Table S1 — (DOCX) [file pone.0074069.s001.docx]

**Table S1. Optical density and percentage of live, compromised and dead bacteria populations after 3, 5 hours and overnight culture at different pH conditions and LBK media.**

| **Growth** |  |  |  | **3h** | | | |  | **5h** | | | | |  | **ON** | | | | |
| --- | --- | --- | --- | --- | --- | --- | --- | --- | --- | --- | --- | --- | --- | --- | --- | --- | --- | --- | --- |
| **conditions** |  | **Strain** |  | **OD_600_** | **Live** | **Com.** | **Dead** | **OD_600_** | | **Live** | **Com.** | **Dead** | **OD_600_** | | | **Live** | **Com** | **Dead** |  |
| **pH 5** |  | E2863wt |  | 1.80±0.27 | 97.6 | 2.1 | 0.3 | 3.35±0.40 | | 99.3 | 0.6 | 0.1 | 5.17±0.65 | | | 99.2 | 0.7 | 0.1 |  |
|  |  | E2863∆CRP |  | 0.48±0.07 | 98.5 | 1.2 | 0.3 | 1.11±0.34 | | 98.8 | 1.1 | 0.2 | 3.82±0.58 | | | 99.3 | 0.4 | 0.3 |  |
| **pH 7** |  | E2863wt |  | 0.56±0.25 | 75.7 | 24.2 | 0.1 | 2.47±0.95 | | 97.7 | 2.2 | 0.1 | 6.13±0.48 | | | 99.4 | 0.4 | 0.2 |  |
|  |  | E2863∆CRP |  | 0.22±0.09 | 96.5 | 3.4 | 0.2 | 0.78±0.41 | | 99.2 | 0.8 | 0.0 | 4.08±0.38 | | | 99.6 | 0.3 | 0.1 |  |
| **pH 9** |  | E2863wt |  | 1.38±0.46 | 4.4 | 94.6 | 0.9 | 3.18±0.38 | | 93.8 | 6.1 | 0.1 | 5.52±0.61 | | | 97.6 | 2.2 | 0.2 |  |
|  |  | E2863∆CRP |  | 0.38±0.06 | 58.9 | 38.6 | 2.5 | 0.94±0.26 | | 92.8 | 7.1 | 0.0 | 3.55±0.53 | | | 85.5 | 14.0 | 0.5 |  |
| **LBK** |  | E2863wt |  | 0.38±0.25 | 97.9 | 1.8 | 0.2 | 1.69±0.51 | | 99.1 | 0.8 | 0.0 | 4.73±1.81 | | | 86.6 | 13.1 | 0.3 |  |
|  |  | E2863∆CRP |  | 0.17±0.10 | 97.9 | 1.9 | 0.2 | 0.40±0.23 | | 99.6 | 0.3 | 0.1 | 2.89±3.38 | | | 99.3 | 0.5 | 0.2 |  |

Com (Compromised).

Measurement of the OD_600_ values and the percentage of live, compromised and dead bacteria are the mean values of 3 independent experiments.
