# Supplementary material for: Alkaline pH Is a Signal for Optimal Production and Secretion of the Heat Labile Toxin, LT in Enterotoxigenic Escherichia Coli (ETEC)
Source: PLoS One. 2013 Sep 18;8(9):e74069. doi: 10.1371/journal.pone.0074069 (PMC3776858; doi:10.1371/journal.pone.0074069)
Supplement: Table S2 — (DOCX) [file pone.0074069.s002.docx]

**Table S2. Primers used in the construction of the ∆CRP mutant strains.**

| **Primer** | **Sequence** |
| --- | --- |
| crp1F | cgcactagttaacccttcgacccacttca |
| crp2R | cccatccactataaactaacagtctgtttgcggtttgc |
| crp3F | tgttagtttatagtggatgggaaaaccatcgtcgtttacgg |
| crp4R | cgcactagttaatccggtcagcaaaaagg |
| crp1F’ | caaccacccgccgccatcaa |
| crp2F’ | cggtttgccaagcaccatgcg |
| crp3R’ | aaaaccatcgtcgtttacgg |
| crp4R’ | taatccggtcagcaaaaagg |
